# Long-term result of posterolateral fusion of the lumbar spine using the Tadpole system

**DOI:** 10.1186/1749-799X-9-33

**Published:** 2014-05-12

**Authors:** Kriangkrai Wittayapairoj, Zhuo Wang, Toshihiko Sakakibara, Yuichi Kasai

**Affiliations:** 1 Department of Spinal Surgery and Medical Engineering, Mie University Graduate School of Medicine, 2-174 Edobashi, Tsu city, Mie 514-8507, Japan; 2 Department of Orthopaedics, Faculty of Medicine, Khon Kaen University, Khon Kaen 40002, Thailand

**Keywords:** Adjacent segment disease, Lumbar degenerative disease, Lumbar spine, Spinal fusion, Spinal instrumentation

## Abstract

**Background:**

Failure of pedicle screw fixation is often seen in patients with severe osteoporosis. We developed new lumbar spinal instrumentation (Tadpole system) for elderly patients who have osteoporotic bone and poor general health status. The objective of this study was to document the long-term clinical outcomes after Tadpole system fixation, the rate of spinal fusion, the incidence of adjacent segment degeneration, the rate of instrumentation failure, and the overall complications.

**Methods:**

Sixty patients who underwent posterolateral spinal fusion using the Tadpole system, in whom a radiograph of the lumbar spine was taken at more than 5 years after operation, were involved in this study. The improvement rate of the Japanese Orthopaedic Association (JOA) score, rate of spinal fusion, presence or absence of adjacent segment degeneration, rate of instrumentation failure, and postoperative complications of each patient were assessed at 5 years postoperatively.

**Results:**

The mean JOA score improvement was 72.5%, and the posterolateral spinal fusion rate was 93.3% (56 of 60 patients) at the last follow-up. Adjacent segment degeneration occurred in only two patients who showed decreased intervertebral disc height, and instrumentation failure (hook deviation) was observed in one patient. No other complications were observed in any patients.

**Conclusion:**

Tadpole system fixation shows favorable long-term clinical outcomes.

## Introduction

**Figure 1:**
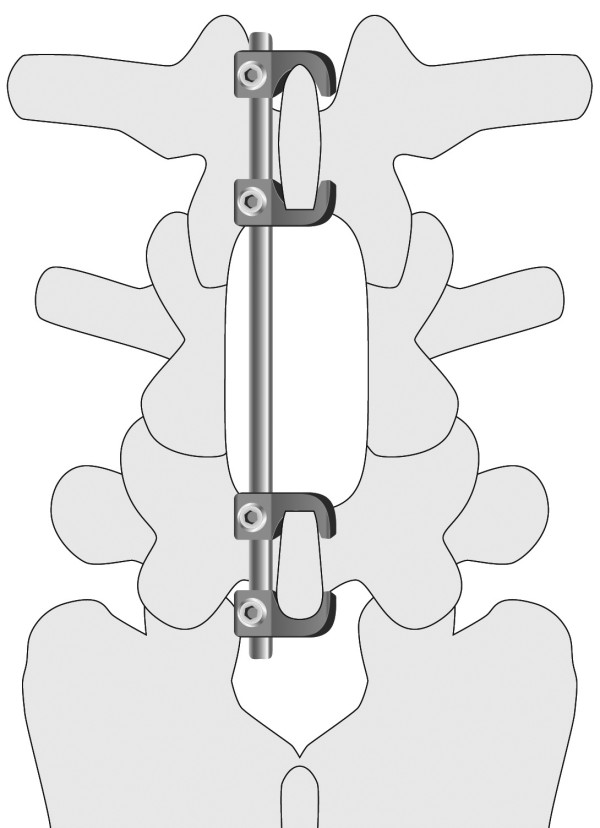
**Tadpole system.** This new spinal instrumentation fixes the spinal process with hooks and rod.

In the last 20 years, the pedicle screw system as part of fixation for lumbar fusion has had good clinical results [1-3]. In a rapidly aging society, a large number of lumbar spinal surgeries are being performed in elderly patients with degenerative diseases, but failure of pedicle screw fixation is often seen in patients with severe osteoporosis [4,5]. We therefore developed new lumbar spinal instrumentation, the ‘Tadpole system’ (KiSCO Co., Ltd., Kobe, Japan, Figure 1), for elderly patients who have osteoporotic bone and poor general health status, who may have a high risk for complex surgeries, and who do not desire a higher activity level in daily life [6]. The Tadpole system fixes the spinous process with four hooks and one rod, and it was commercialized in 2004. Moreover, we showed that the operative procedure with this system is easy, and we reported good short-term clinical results [6].

In the present study, the long-term clinical outcomes in patients who have used the system for more than 5 years were investigated. The objective of this study was to document the long-term clinical outcomes after Tadpole system fixation, the rate of spinal fusion, the incidence of adjacent segment degeneration, the rate of instrumentation failure, and overall complications.

## Materials and methods

Of the 74 patients in whom the Tadpole system was used from 2004 to 2008, 9 patients died and 5 patients were lost to follow-up (change of residence in 1 patient and unknown in 4 patients). Therefore, 60 patients (25 males, 35 females) for whom a radiograph of the lumbar spine was taken at more than 5 years after operation were included. A key inclusion criterion for this study was age 75 years or older. These patients represent a population that pursues less demanding daily activities. In these patients' activities of daily living, their performance status, as an evaluation of their preoperative general condition by the Eastern Cooperative Oncology Group, was grade 0 in three patients, grade 2 in two patients, and grade 1 in the remaining patients, and all patients could have a nearly self-organized daily life. The diagnoses were lumbar spinal stenosis in 47 patients and spondylolisthesis in 13 patients. The levels of fixation of the intervertebral disc space were L2-4 in 5, L3-5 in 48, L3-4 in 2, and L4-5 in 5 patients. The exclusion criteria were Meyerding classification grade 2 or more spondylolisthesis, obvious degenerative scoliosis, and patients who required lumbar interbody fusion as a consequence of their need to pursue physically demanding activities after the operation.

Since leg pain and intermittent claudication were observed in all patients, central and lateral canal decompression with ipsilateral and contralateral neural foraminotomy was performed. In our institution, if intervertebral instability was not observed on the imaging related to the treatment of lumbar vertebral degenerative disease, laminotomy was performed while preserving the facet as much as possible, and if instability according to the following definition was observed on imaging, posterolateral fusion in addition to laminotomy was performed using the local bone obtained at decompression. Moreover, the spinal instrumentation was combined; the Tadpole system was used for the elderly aged 75 years or older, and the pedicle screw system was used for the elderly aged less than 75 years. In this institution, surgery for patients with lumbar vertebral degenerative disease included laminotomy alone, the Tadpole system, and the pedicle screw system in proportions of about 50%, 10%, and 40%, respectively, from 2004 to 2008. Intervertebral instability was defined as the presence of at least one of the following three findings on radiography of the lumbar vertebra: (1) angular motion 20° or greater, (2) translational motion 5 mm or greater, and (3) intervertebral endplate angle in the flexion film minus 5° or less. For the postoperative protocol, the patients were instructed to use a lumbar brace for 3 months postoperatively.

The clinical outcome parameter consisted of the JOA score (Japanese Orthopaedic Association scoring system for lumbar spinal disorders, full marks of 29 points). This includes pain, walking ability, muscle strength, neurological findings, activities of daily living, and bladder function, which were evaluated by the doctor before operation and at the last follow-up. The improvement rate in the JOA score (%, Hirabayashi method), the rate of spinal fusion, the presence or absence of adjacent segment degeneration, the rate of instrumentation failure, and postoperative complications at the last follow-up 5 years postoperatively were evaluated.

Adjacent segment degeneration was defined on the plain film as accelerated degenerative changes at the adjacent level on the cranial and caudal sides of fixed vertebrae, using the following criteria: (1) more than 50% narrowing of the height of the intervertebral disc at the adjacent level of fixed vertebrae compared with that before operation, (2) occurrence of fresh spondylolisthesis of 3 mm or more at the adjacent level, (3) posterior wedging angle of 5° or more at the adjacent level under forward flexion motion, and (4) new compression fracture at the adjacent vertebrae. All of these were evaluated with radiographs taken in the upright position [7]. If any one of (1) to (4) was observed, the patient was considered to have adjacent segment degeneration. The height of the intervertebral disc was measured as follows: (Height of anterior border of intervertebral disc + Height of posterior border of intervertebral disc)/2.

In the evaluation of spinal fusion, if at least one of (1) obvious continuity of trabecula between fixed vertebrae on the radiographs of lumbar vertebrae and (2) intervertebral range of motion 2° or less on the dynamic lateral plain film was present, the patient was considered to have spinal fusion [8,9]. The presence or absence of adjacent segment degeneration and spinal fusion was evaluated by two independent observers (orthopedic surgeon A with 20 years of experience and orthopedic surgeon B with 7 years of experience). If the results of the evaluation were not consistent between the two observers, they were evaluated by a third independent observer (orthopedic surgeon C with 27 years of experience). The consistency of the evaluations performed by orthopedic surgeons A and B was evaluated with the kappa (*κ*) coefficient. This study was performed with the approval (No. 2662) of the ethics committee of our university.

### Statistical analysis

Descriptive continuous data are reported as means ± SD (range). Categorical data are reported as percent (%). The kappa (*κ*) coefficient was used to examine agreement between observers.

## Result

**Figure 2:**
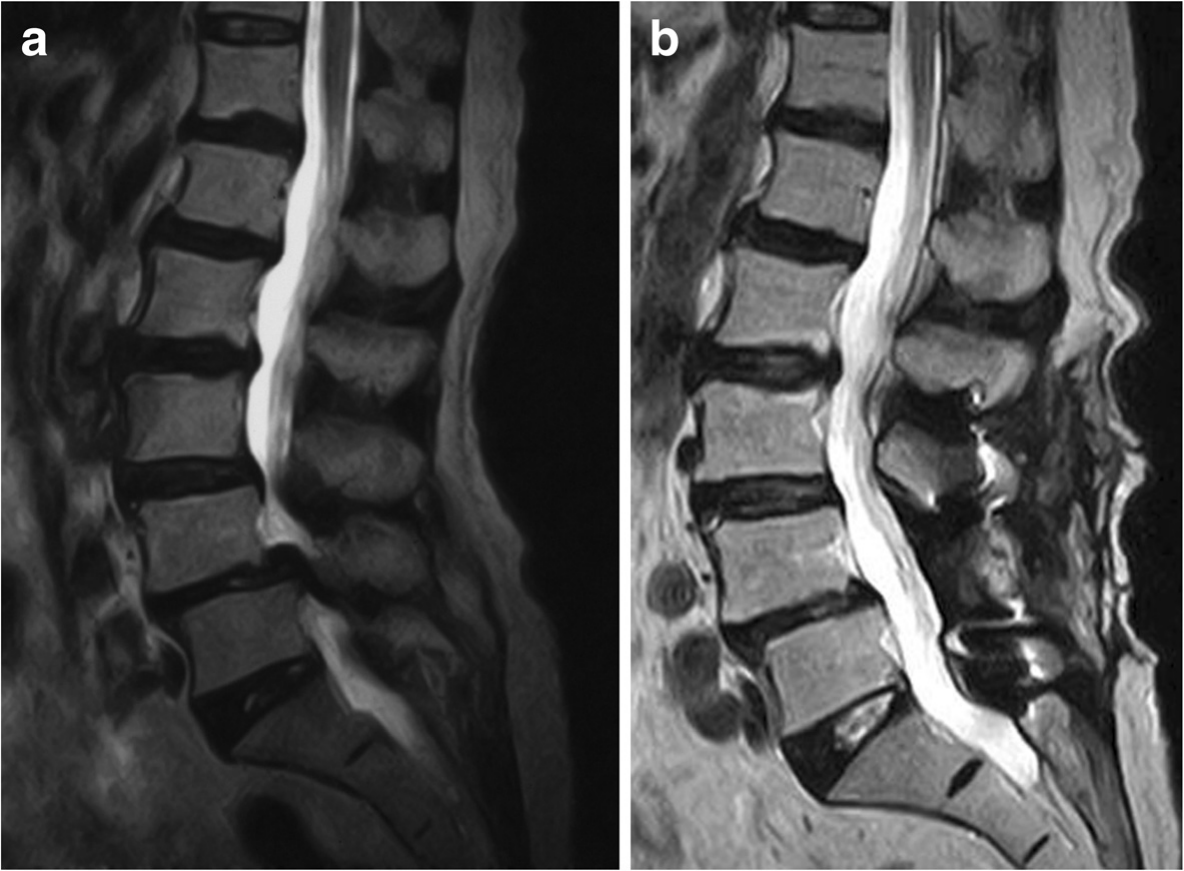
**MRI of a 76-year-old female with spondylolisthesis. (a)** Preoperative T2-weighted image. **(b)** Postoperative T2-weighted image.

**Figure 3:**
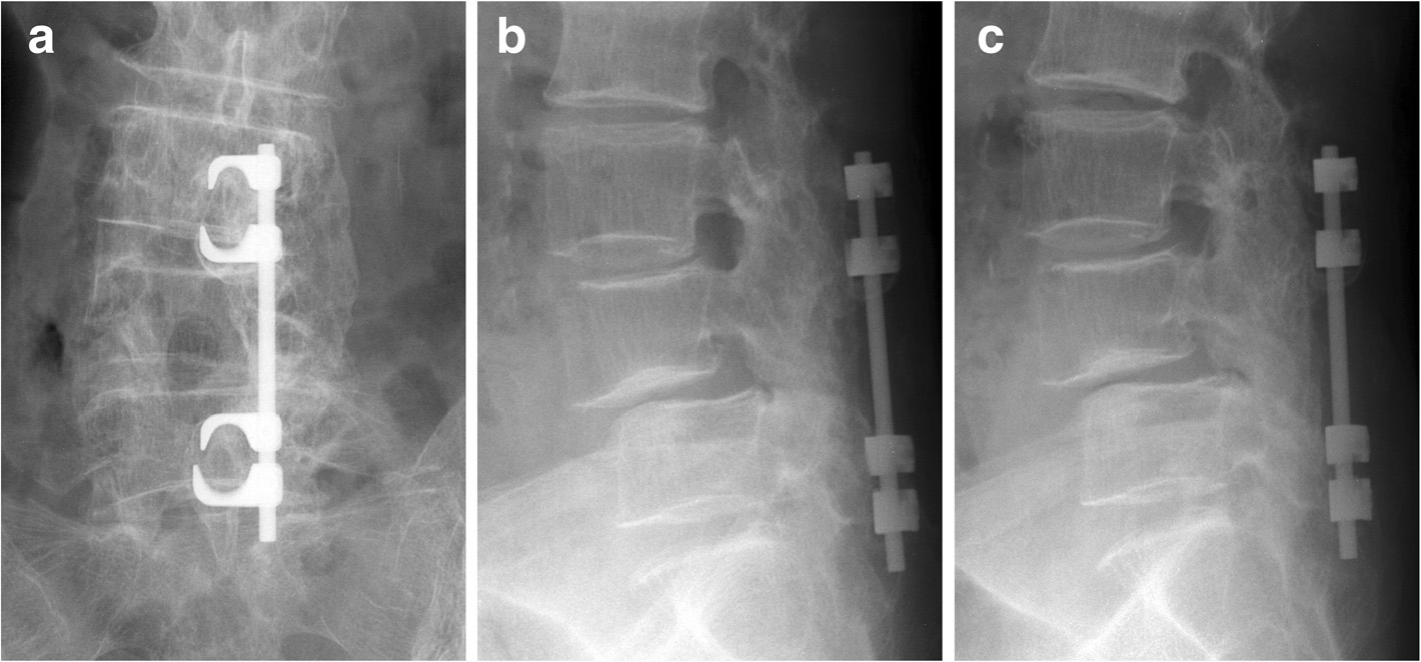
**Preoperative X-rays 5 years after surgery of an 80-year-old female.** The X-rays show a good fusion mass and no evidence of adjacent segment degeneration. **(a)** Anteroposterior view. **(b)** Lateral view of anteflexion. **(c)** Lateral view of retroflexion.

The patients' mean age at operation was 84.2 years (range 75–96 years), and the mean postoperative follow-up time was 80 months (range 60–109 months). The mean JOA score was 11.9 ± 6.3 points before operation and 24.3 ± 5.7 points at the last follow-up; the mean postoperative improvement was 72.5% ± 26.2% (Figure 2a,b). Posterolateral spinal fusion (Figure 3a,b,c) at the last follow-up was achieved in 56 of 60 patients (93.3%), and of the 4 patients who did not achieve spinal fusion, 1 had moderate low back pain and the other 3 had no symptoms. Adjacent segment degeneration occurred in only two patients showing decreased intervertebral disc height. No patients developed obvious low back pain or neurological symptoms. The agreement between observers was high (*κ* = 0.88). With regard to instrumentation failures, hook deviation was observed in one patient in whom spinal fusion was completed, with no low back pain or neurological symptoms. Postoperative complications, such as cerebrospinal fluid leak, nerve paralysis, postoperative infection, or spinous process fracture, were not observed in any patients.

## Discussion

Spinal instrumentation using the spinous process as an anchor has been used for about 50 years (Daab plate or Wilson plate) [10,11], but in view of the inferior biomechanical strength, not many instrumentation systems continue to use a spinous process anchor. In recent years, relatively good clinical results of lumbar spine fixation have been reported with the Lumbar Alligator Spinal System, CD HORIZON SPIRE spinous process plate and S-plate, as spinal instrumentation using a spinous process as an anchor [12,13], but the Tadpole system is a unique spinal instrumentation hook and rod system. Shepherd et al. [14] demonstrated that the spinous process had sufficient grasping capacity when a hook was placed in the spinous process, suggesting the validity of fixing our Tadpole system to the spinous process with a hook.

The Tadpole system is more suitable for elderly patients who have osteoporotic bone and poor general health status and may be at high risk for complex surgeries. The authors have already reported 2-year follow-up clinical results on the Tadpole system in 31 degenerative lumbar spine patients in 2008 [6]. A mean 70.9% improvement rate in the JOA score and 93.5% fusion rate with no major complications were obtained, which showed that this system might be a useful, easy-to-use, and safe spinal instrumentation technique for lumbar fusion surgery.

In the present long-term follow-up study, the mean improvement rate in the JOA score was 72.5%, and the fusion rate of posterolateral fusion was 93.3%, showing a good clinical outcome. Based on the reports from the literature, the improvement rate in the JOA score after pedicle screw fixation was 66.9%–84.1%, and the posterolateral fusion rate was 68%–100% [15-21]. For the fusion rate and the functional outcome, the Tadpole system showed no difference when compared with pedicle screw fixation. With respect to biomechanical data, the stability of the Tadpole system is certainly inferior to that of the pedicle screw system, but it is more likely to achieve bone fusion than non-instrumentation. Furthermore, intervertebral disc degeneration of elderly patients aged 75 years or older has already progressed, and, moreover, the patients' postoperative activity levels were low. This was considered to be the reason for the good bone fusion in the present study.

With respect to intra- and postoperative complications, cerebrospinal fluid leakage, nerve injury, deep infection, and instrumentation failure have been reported in 4%–10%, 2%–5%, 4%–5%, and 3%–12% of patients, respectively [20,22,23]. In the present study, hook deviation occurred in one patient as a case of instrumentation failure, but no other major complications were observed.

With respect to adjacent segment degeneration, Ghiselli et al. [24] reported that 59 (27.4%) of 215 patients had degeneration at an adjacent segment after posterior lumbar arthrodesis with the pedicle screw system. Lund and Oxland [25] reported that the rate of adjacent segment degeneration in the literature varied from 11% to 100%. Park et al. [26] reviewed the literature and concluded that the incidence rate of adjacent segment disease ranged from 5.2% to 18.5% over 44.8 to 164 months of follow-up observation. The rate of adjacent segment disease is higher in patients with pedicle screw spinal fixation (12.2%–18.5%) than in patients with no instrumentation (5.2%–5.6%). In the present study, the incidence of adjacent segment degeneration was only 3.3%. It is possible that stress or torque at the adjacent segment level may be lower in Tadpole system fixation than in pedicle screw system fixation, and alternatively, a lower rate of adjacent segment disease may be no disadvantage of the rigid pedicle screw system rather than an advantage of the Tadpole system. A possible selection bias exists in choosing patients for the Tadpole system. These patients have low physical demand in their activities of daily living. From the above, it was considered that adjacent segment degeneration with the Tadpole system might hardly ever occur, and further biomechanical studies are needed to identify the reason.

The limitations of the present study are that it was a retrospective case series without a control group. There was also a bias, in that old and less active patients were selected as patients for the present study. The authors suspect that it is very difficult to compare clinical data for the Tadpole system with that for the pedicle screw system, because there are significant differences between elderly patients treated with the Tadpole system and young and active patients treated with the pedicle screw system. However, a prospective, age-matched, randomized, controlled trial may be necessary to investigate the real clinical significance of the Tadpole system. Another limitation of this study is that there were no data for bone mineral density, an issue which the authors will continue to consider.

## Conclusion

The long-term results of posterolateral fusion of the lumbar spine using the Tadpole system for old and less active patients were good, with a mean JOA score improvement of 71.5% and a posterolateral spinal fusion rate of 93.3% at the last follow-up. Adjacent segment degeneration occurred in only two patients showing decreased intervertebral disc height, and deviation of a hook (instrumentation failure) was observed in one patient. No other complications were observed in any patients. The Tadpole system is a useful spinal instrumentation system with favorable long-term clinical outcomes in patients with moderate to low physical demands.

## Competing interests

No funds were received in support of this work. No benefits in any form have been or will be received from a commercial party related directly to the subject of this manuscript. The corresponding author is a producer of the Tadpole system. The other authors declare that they have no competing interests.

## Authors' contributions

KW drafted the manuscript, did the first selection of articles, and assessed the quality of the papers. ZW and TS gave important input for the methods part of this paper, assessed the quality of the papers, performed the statistical analysis, and revised the manuscript critically for its content. YK helped to draft and correct the manuscript. All authors read and approved the final manuscript.
